# Microcantilever-based current balance for precise measurement of the photon force

**DOI:** 10.1038/s41598-022-27369-3

**Published:** 2023-01-10

**Authors:** Bartosz Pruchnik, Karolina Orłowska, Bartosz Świadkowski, Ewelina Gacka, Andrzej Sierakowski, Paweł Janus, Teodor Gotszalk

**Affiliations:** 1 Department of Nanometrology, Faculty of Electronics, Photonics and Microsystems, Wrocław University of Science and Technology, Janiszewskiego 11/17, 50-372 Wrocław, Poland; 2 Institute of Microelectronics and Fotonics, Łukasiewicz Research Network, Lotników 32/46, 02-668 Warsaw, Poland

**Keywords:** Engineering, Nanoscience and technology, Optics and photonics

## Abstract

We present a method for the quantitative determination of the photon force (PF)—the force generated by the radiation pressure of photons reflected from the surface. We propose an experimental setup integrating innovative microelectromechanical system (MEMS) optimized for the detection of photon force (pfMEMS). An active microcantilever was used as the force detector, while the measurement was conducted in a closed-loop setup with electromagnetic force compensation. In opposition to our previous works, this measurement method provides quantitative not qualitative assessment of PF interaction. Final current-balance setup is suitable for light sources from tens of microwatts to few watts. In our article, we present the results of the performed experiments, in which we measured the PF interactions in the range up to 67.5 pN with resolution of 30 fN in the static measurement.

## Introduction

Nanometrology, defined as the quantitative description of nanoscale phenomena, seeks specific metrological calibration standards. For this purpose, precise microelectromechanical systems (MEMS) are being constructed^1^ employing a series of techniques for deflection actuation and deflection detection^2,3^. With the use of nanometrological devices, it is possible to measure distances down to femtometers^4^ and forces down to femtonewtons^5,6^—the order of magnitude at which photon force (PF) is measurable.

Force induced on the surface by light beam was predicted in the nineteenth century by Maxwell and Bartoli in the theoretical descriptions of electromagnetic waves. PF was not proven in experiments until the invention of the Nichols radiometer in 1901^7^. Since this presentation, for over a century, different methods of PF measurements have been created, including torsion balance^8^, electrostatic^9^ and piezoelectric approaches^10^.

There are many applications based on the PF phenomenon. The most recognizable technologies are manipulations of fine particles in optical tweezers^11^, and the force induced by photons is also considered a potential propulsion drive in space solutions^12^.

Today’s technology makes it possible to generate light beams with a power in the range from pW up to PW (10^15^ W). Optomechanical phenomena are the subject of high-resolution and traceable metrology. This is of the highest interest for high and low energies, as in both cases, the stream of photons can be used to actuate the displacement of the mechanical systems and devices.

At the nanoscale, the PF interaction can exert force on the structure with extremely high force resolution. PF may be precisely electronically controlled with a well-characterized radiation source and even better in a back-action setup. In this case the PF actuated devices can be used in various environments. However, in the metrological solutions the force must be calibrated. Thus is the requirement to design metrological devices to measure PF interactions with high resolution and repeatability.

From the historical point of view a current balance was used to determine the equilibrium state between the gravity of an object and the electromagnetic force of an electromagnet. In the current balance, as indicated by its name, gravity force is expressed by the electrical quantities of current and voltage. The sensitivity of the current balance is limited by the sensitivity of the displacement detectors. This creates a metrological device and the possibility to express forces (originally weight) with electrical quantities. In this setup, even very fine forces can be detected, as the sensitivity is determined by the mechanical properties of the balance and sensitivity of the displacement sensor.

MEMS are tools frequently used in measurements of fine forces, therefore they were used also in measurements of PF interactions. Approaches cover the use of a microcantilever as a force–deflection transducer^13^, active compensation of deflection was also introduced^14^. However, to our best knowledge there is no work introducing a MEMS setup with direct force compensation of measurement of PF.

Approaches utilizing current balance for measurement of photon force were already introduced with macroscopic coupled mirror setup^15,16^. Resolution in these setups was limited by the effects coupled with dimensions of the setup and did not exceed 20 nN lower limit. Therefore miniaturization of the force compensation device should bring improvement in the range of detectable forces.

In this article we present, in our opinion for the first time, a PF metrological MEMS (pfMEMS) devices operating as a current balance. The heart of the system is a silicon microcantilever, whose U-shaped legs are conductive in volume. Moreover, it integrates a micromirror on which the analyzed photon beam is focused. The detection of the microcantilever deflection is done using an optical beam detector (OBD) serving as a zero indicator. The microcantilever is immersed in the magnetic field, thus when the current flowing through the U-shaped legs is controlled, it is possible to compensate the PF interactions. The described procedure can be performed in resonance and static mode with resolution of 30 fN corresponding with the optical power of 5.9 µW.

## Results

**Figure 1:**
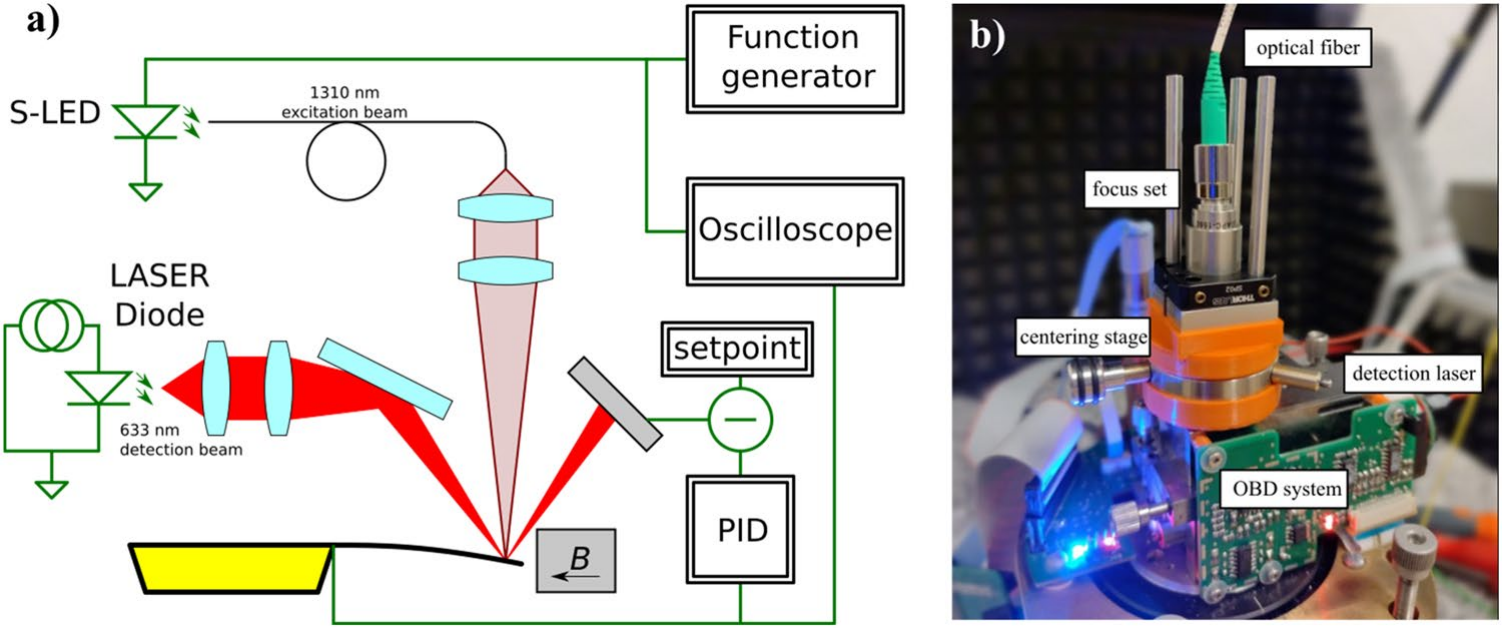
Experimental setup: (**a**) schematic of the setup with electrical and optical signals; (**b**) optical part of the setup.

The experimental setup (Fig. 1a) consists of a pfMEMS mounted in a specially prepared holder with an embedded magnet exciting stable magnetic field. In the closed loop an OBD system and a dedicated PID controller were applied^17,18^. To focus the actuation beam on the mirror of the microcantilever, the following optical setup was used: a Thorlabs S5FC1018P SLD light source, a Thorlabs P3-1064Y-FC-1 single mode patch fiber for the second transmission window, a Thorlabs F230APC-1550 collimation lens and an Edmund Optics 6.25 mm diameter and 60 mm focal length focusing doublet lens. Additionally, a signal generator AFG 3021B and a TDS 1004B multichannel oscilloscope were used to define control signals and observe the adjustment of the optics.

To ensure the proper position of the laser beam on the microcantilever, a specialized stage was produced (Fig. 1b). The developed stage makes it possible to adjust the height of the stage, so that the distance was equal to the focal length of the focus lens. The horizontal position of the spot was set manually with the Elliot Martock MDE250S-15 centering mount. Correct positioning was achieved with a HeNe laser. The spot was observed optically on the microcantilever and was set to be on the center of the pfMEMS mirror.

The size of the spot was determined by the characteristic of the optical setup. The beam is first collimated and then focused with convergence *θ*. Calculations were conducted for wavelength *λ* = 1310 nm and Gaussian profile of the beam. The calculated diameter of the spot was:

1 $$d=\frac{2\lambda }{\theta \times \pi }=\frac{2\times 1.3}{0.017\times \pi }\approx 49 \, \upmu\text{m}.$$

The diameter of the golden mirror is 40 μm. The calculation of energy taking into account the Gaussian profile of the beam shows that a beam focused precisely on the mirror delivers 75% of power directed to the surface of the mirror.

The Thorlabs S5FC1018P SLED light source allows for the external modulation of the beam intensity. In the performed experiment, the microcantilever was actuated with a photon beam of the power ranging from 0 to 45.71 mW, as measured by a bolometric sensor. To further eliminate thermal influences, the measurements were conducted with a light beam directed onto the microcantilever from its both sides—from the top and bottom. Thermal actuation is insensitive to the direction of the force, in contrary to the electromagnetic force and photon force. The results obtained with both methods should differ by the margin of thermal deflection.

The output signal of the PID controller was recorded by a digital oscilloscope. The bias current flowing through the microcantilever was determined according to Ohm’s law as the relation of the voltage signal and the resistance of the actuation loop. The resistance of the microcantilever loop with the current limiting resistor connected in series was 60.2 kΩ (the resistor and microcantilever resistances were 56 kΩ and 4.2 kΩ, respectively). The product of the B-field and length of the path on the microcantilever was previously calibrated in interferometric measurements (Eqs. (8), (9)), therefore eliminating error of magnetic field measurement from the solution. Calculations of the PF are then traceable back to the wavelength of the interferometer laser and measurement of actuation current.

**Figure 2:**
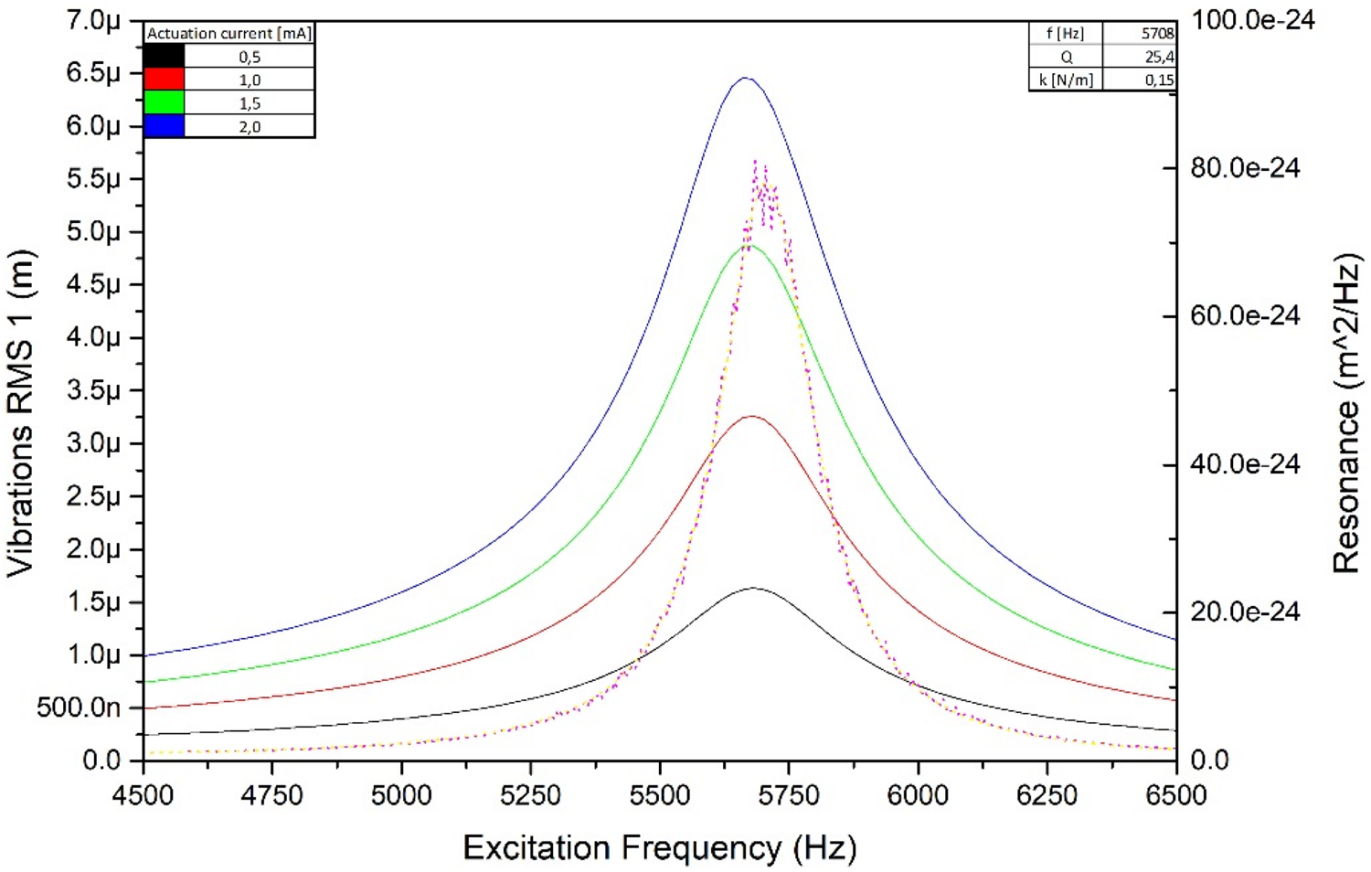
Set of the microcantilever characteristics: actuation curves (continuous lines) for a various actuation currents, the thermomechanical noise characteristics presented as a reference.

In the first step the microcantilever was actuated with a series of currents from 0 to 2 mA with a 0.5 mA step (Fig. 2).

In the second step evaluation of the force resolution of the setup was done. Resolution limitation is noise level of the OBD setup. Noise was estimated with root mean square error (RMSE) measurements of steady regulation in which microcantilever deflection was held at given value. RMSE value was measured to be 0.137 mV, which corresponds with 30.8 fN of force and optical power of 5.9 μW.

In the third step a range of the measurement was estimated. It had been proven experimentally that RMS input electrical power of 50 mW is an absolute critical value for pfMEMS, which corresponds to the 3.45 mA of RMS of current signal. This current value corresponds with compensating force 46.6 nN and optical power 8.9 W.

**Figure 3:**
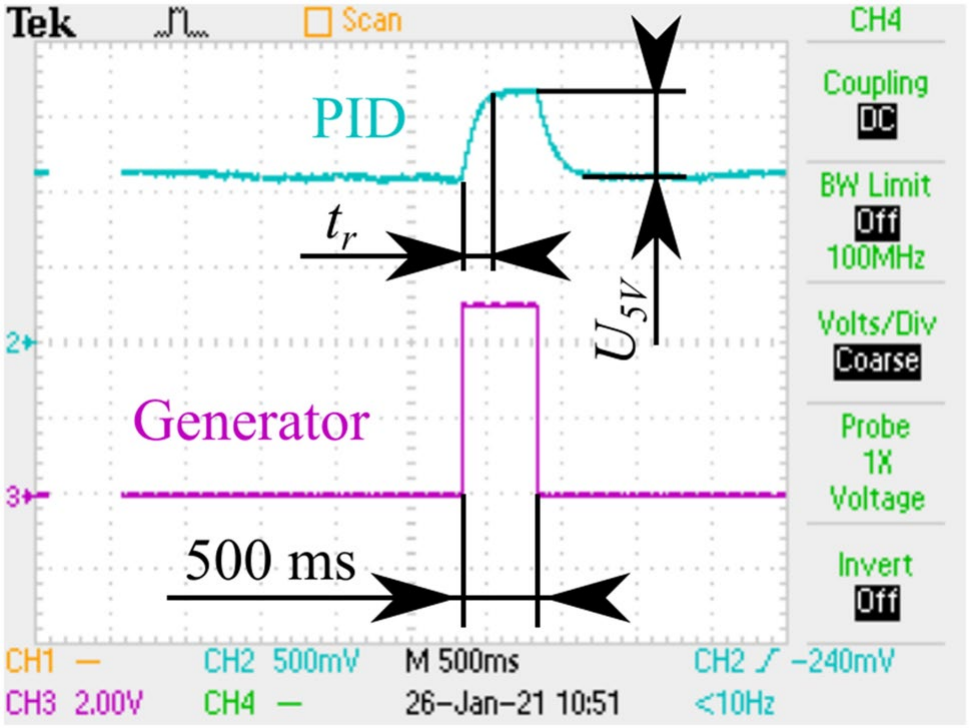
Control behavior of the electromagnetic microcantilever during PF measurement.

Finally, the pfMEMS was actuated with a laser beam. To eliminate various drifts and low-frequency noises, the laser beam was controlled with the pulse signal with the defined amplitude of a selected voltage, period of 5 s and pulse width of 500 ms. Pulse width was set long enough to avoid influence of the regulation Time constant. Time of regulation was presented in registered characteristics as *t*_*r*_ (Fig. 3).

For 5 V of actuating signal, the SLD emits a power of 45.71 mW. The recorded signals for the microcantilever actuated from above (top actuation) and from below (down actuation) are equal to ${U}_{5 \, \text{V}}^{up}=0.610 \, \text{V}$ and ${U}_{5 \, \text{V}}^{down}=0.563 \, \text{V}$, respectively. Given that the resistance of the setup equals *R* = 60.2 kΩ, the measured *PF* was:

2 $${PF}_{5\text{ V}}^{up}=\frac{{U}_{5\text{ V}}^{up}}{R}Bl=\frac{0.610 \text{ V}}{60.2 \text{ k}\Omega} \times 0.135\, \text{ mT}\times 100 \,\upmu\text{m}\approx 10.13 \,\upmu\text{A}\times 0.135\, \text{mT}\times 100 \,\upmu\text{m}\approx 137 \text{ pN}.$$

3 $${PF}_{5\text{ V}}^{down}=\frac{{U}_{5\text{ V}}^{down}}{R}Bl=\frac{0.563 \text{ V}}{60.2 \text{ k}\Omega} \times 0.135\, \text{mT}\times 100 \,\upmu\text{m}\approx 9.35 \,\upmu\text{A}\times 0.135\text{ mT}\times 100 \,\upmu\text{m}\approx 127 \text{ pN}.$$

Exact values for each setpoint are presented in Table 1. The difference in the asymmetry of the calculated force signs in the setup is caused by the ongoing thermal deflection. The deflection of the pfMEMS is proportional to the force; thus, the deflection difference is expressed by the force difference. The real photon force is equal to the mean of the $P{F}^{up}$ and $P{F}^{down}$ values and therefore to $PF = 132 \text{ pN}$.

**Table 1:** Measured photon forces and actual differential signals present in the setup.

U_exc_ (mV)	Top actuation			Down actuation			Photon force (mW)
	U (mV)	F (pN)	P (mW)	U (mV)	F (pN)	P (mW)	
1500	80	18	3.90	66	15	3.22	3.56
2000	207	46	10.09	147	33	7.17	8.63
2500	301	67	14.67	257	58	12.53	13.60
3000	369	83	17.99	352	79	17.16	17.57
3500	477	107	23.25	440	99	21.45	22.35
4000	524	118	25.54	494	111	24.08	24.81
4500	549	124	26.76	542	122	26.42	26.59
5000	610	137	29.73	563	127	27.44	28.59

The formula for the calculation of the beam power, which takes into account PF and material, can be written as:

4 $$P=c\frac{PF}{A+dR},$$

which is used to calculate the beam power. If the beam interacts only with the mirror whose parameters can be described with the quantities *A*, *R* and *T* for absorption, reflection and transmission respectively with coefficient *d* describing diffusivity, the power directed on the mirror is:

5 $${P}_{5\text{ V}}=3\times {10}^{8} \, \frac{\text{m}}{{\text{s}}}\times \frac{132\text{ pN}}{0.001+1.78\times 0.99}\approx 25.2 \text{ mW},$$

where its value is lower compared to the earlier measured value of 45.71 mW.

**Figure 4:**
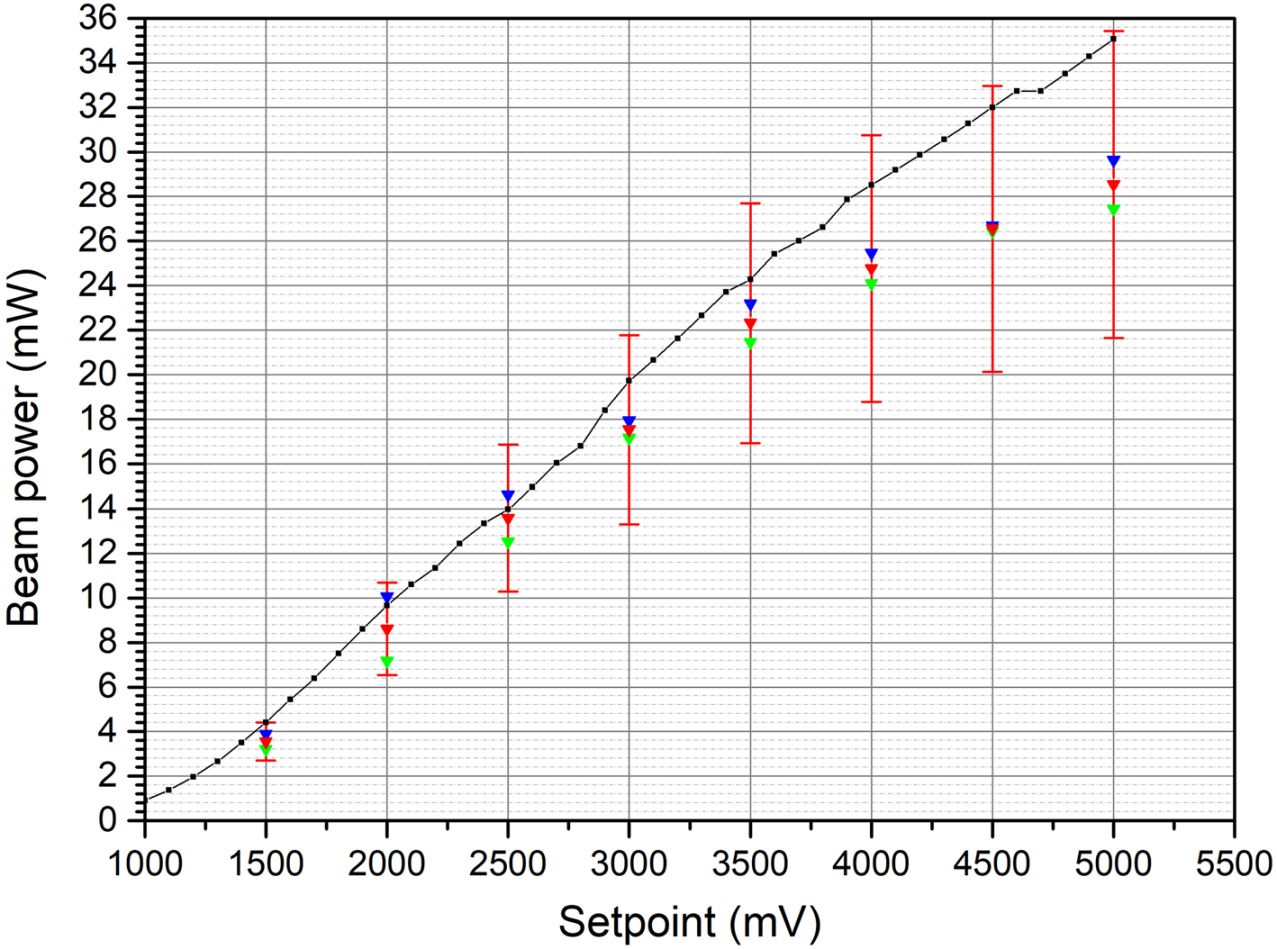
Power of the SLED beam (scatter series) measured with current balance compared with the optical power measured with conventional photodiode (continuous line). Correction factor coming from geometrical parameters was applied. Error bars correspond with measurement error.

The same calculations were conducted for a set of setpoints and the obtained results are presented in Fig. 4.

## Discussion

Presented setup allows measurements of the PF with sensitivity down to 5.9 μW. The obtained results show that the measured PF using the pfMEMS setup is not affected by thermal effects or external noise. The calculated optical power is lower than the reference power of the SLD source, but is contained within range of error. The difference comes from the imperfect collimation of the optical beam on the pfMEMS mirror and imprecise value of the diffusivity of the mirror, which included in the graph as the measurement error. The OBD setup integrated with the pfMEMS was utilized in the current balance setup as a high resolution device for the detection of forces as small as a few piconewtons. Furthermore, it can be applied in nanometrology as a force reference working in the range of up to several nanonewtons. The measured resolution and stability of the device is in the range of tens of femtonewtons, so is the uncertainty of the actual force measurement.

The improvement of the proposed setup will be achieved when the reflective mirror area will correspond with photon beam diameter. Another source of uncertainty is connected with the unknown scattering properties of the thin film integrated mirror. In the performed calculations the dispersive surface of the mirror was considered. Right now measurement error is effectively determined by unknown parameter of diffusivity.

There still exist technological advances which can be introduced. Further elimination of thermal influences would most certainly improve the sensitivity and resolution of the setup. However the obtained results clearly show, that the technology based on the pfMEMS can be used to construct systems in which mass, force of molecular interactions should be measured in the traceable manner.

## Materials and methods

### Photon force

**Figure 5:**
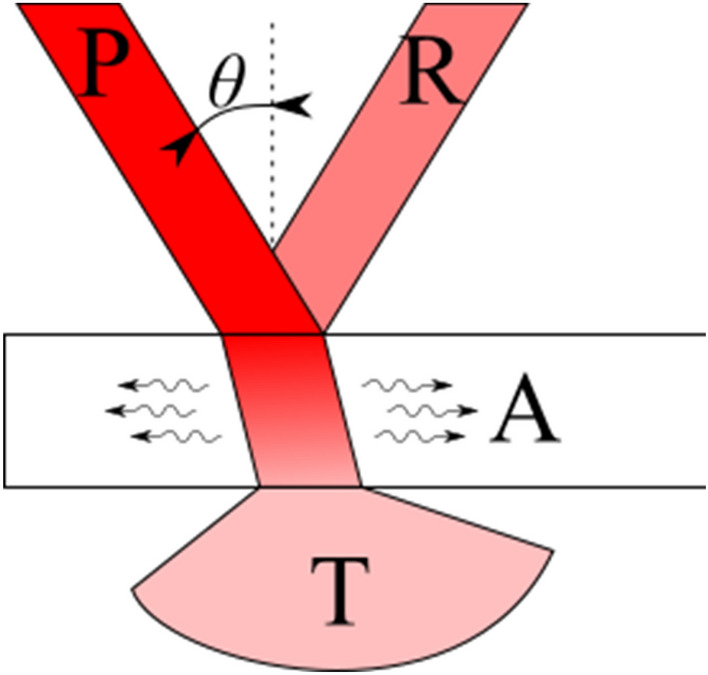
Reflection-transmission-absorption model of the interaction of light and solid matter.

From the theoretical point of view, light exerts a force proportional to the power of the light beam on a mirror surface. Specular reflection transmits to the surface the total momentum of photons ingoing and outgoing onto the surface. When the light beam of power P is directed to the mirror surface, it can be reflected, absorbed and transmitted (Fig. 5). These interaction are quantitatively described with R (for reflection), A (for absorption) and T (for transmission); R, A and T sums up to 100% of P. In this case the force exerted on the mirror can then be described by the following formula:

6 $$\text{PF}=\frac{P\text{cos}\theta \left(dR+A\right)}{c},$$

where *θ* is the incidence angle and d is coefficient deriving from diffusivity of the surface. Purely reflective material will reflect beam with repeated incidence angle; purely diffusive will scatter the light in uniform sphere. Photons are absorbed by the material (S_in_) and then emitted from the opposite side of the bulk (S_out_). It is important to separate these two stages, as scattering can differ in angular distribution (Ω) from absorption; in that case, transmitted light also takes part in the photon force generation. In such a case, the complete equation is^13^:

7 $$\text{PF}=\frac{P\text{cos}\theta \left(dR+A+{S}_{in}-\frac{{S}_{out}\left(\Omega \right)}{\text{cos}\theta }\right)}{c}.$$

The properties of the perfect experiment are: a completely reflective material illuminated with a light beam with zero incidence angle, resulting in numerator equal simply 2 times *P*.

### Optical beam deflection setup

The OBD detector consists of a collimated beam, adjustment components, actuator and position sensitive detector (PSD). The object movements cause the movements of the laser spot on the PSD. Object displacements in the chosen direction are represented by the displacement of the laser spot on the PSD. The position of the spot on the photodetector is determined by the photocurrents of separate segments. The imbalance signal is the input of the feedback loop in which the regulator sets the position of the measured object with the use of an actuator. Information delivered solely by the photodetector is not metrological. The actual measured quantity is derived from the regulator signal controlling the current flowing through the pfMEMS.

The main advantages of the OBD setup are a relatively low level of noise in both static and harmonic actuation and a high sensitivity of deflection detection. The PSD signals are normalized by the total power of the optical signal to eliminate the influence of variations in the optical signal power of the illuminating laser.

Force compensation setup can be realized within an OBD setup. With a proper sensor—a force–deflection transducer—the feedback loop regulation signal represents the force necessary to balance the external force.

### Electromagneticaly actuated microcantilevers

The microcantilever deflection can be easily actuated using external actuators, such as piezoelectric actuators, voice coils, thermal. The more reliable actuation methods involve techniques that excite displacement directly on the micromechanical structure. In this case, electrothermal, piezoelectric, and electrostatic technologies can be applied^19–22^.

**Figure 6:**
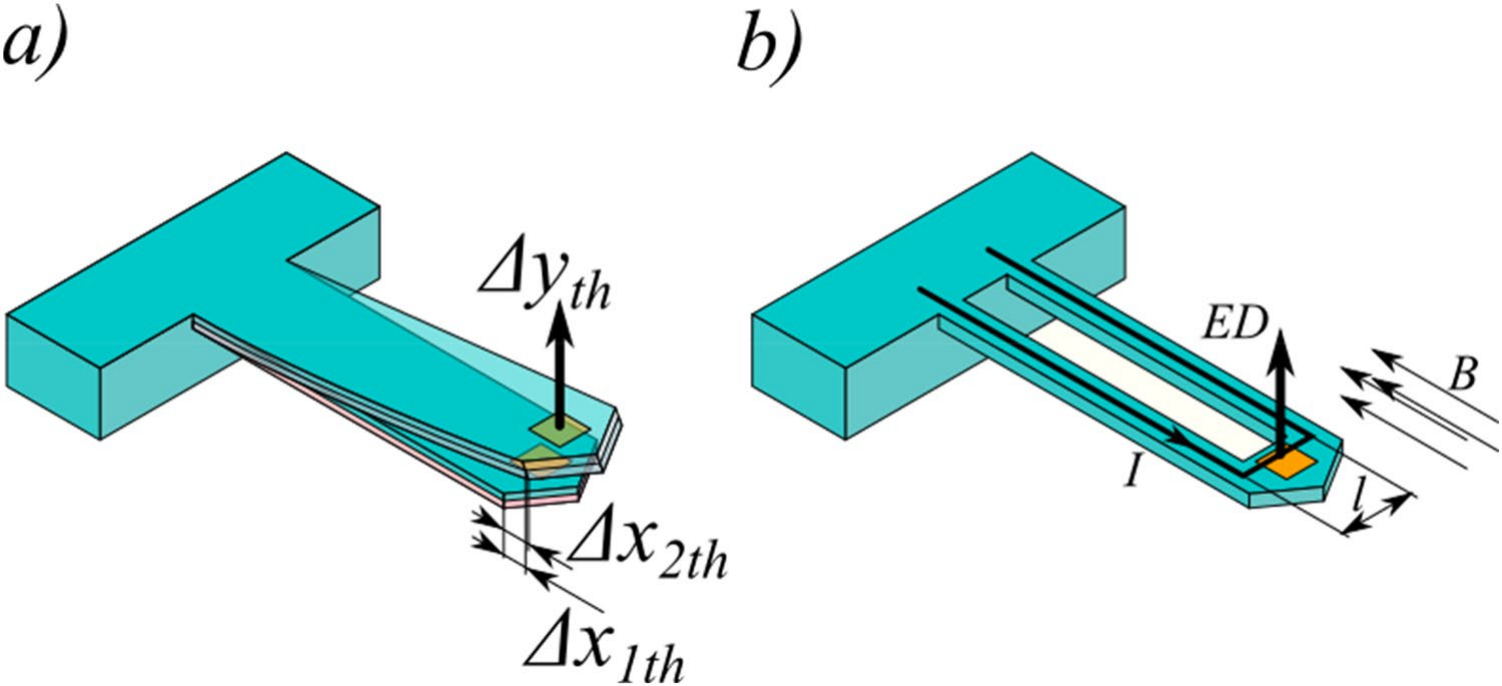
Actuations: (**a**) thermomechanical and (**b**) electromagnetic.

A new kind of microcantilever was presented in our previous work^5^. Its construction was optimized to provide greater force sensitivity to measure forces as small as surface adhesion or radiation pressure. It was made basing on the silicon on insulator (SOI) technology to assure uniform thickness. The cantilever was formed out of a highly doped silicon (10^19^), which resulted in the cantilever’s approximate resistance of few kiloohms. Uniform properties of the material across the microcantilever’s thickness reduced the influence of thermal (multilayer) actuation (Fig. 6a). The pfMEMS microcantilever integrated a gold mirror at the structure end. For wavelengths greater than 650 nm, gold is over 99% reflective^23^. In order to reduce the temperature influence, the mirrors were separated thermally from the main mass of the device by a set of small silicon legs, which on one hand should be stiff but at another their thermal resistance was as high as possible.

In the same work^24^, the authors showed that the detection of photon force is possible with this type of the device. The performed experiment covered the harmonic actuation of the microcantilever with modulated laser light. In following work, the shape of the microcantilever was optimized in terms of force sensitivity^25^.

**Figure 7:**
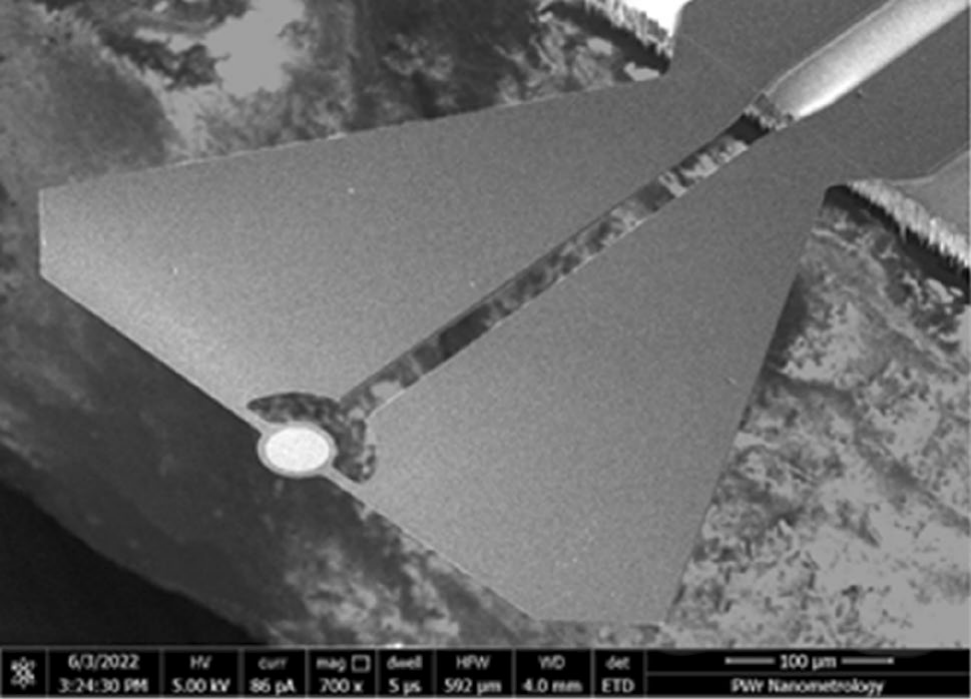
Scanning electron microscope images of a thermally enhanced pfMEMS microcantilever for PF investigation.

In this work, a microcantilever from the next series is presented (Fig. 7). The materials and technology are the same as in the previous series. The microcantilever was optimized in terms of heat flow leading to the temperature distribution along the device and causing parasitic structure deflection.

The microcantilever mass around device mirror is increased forming a thermal capacitor which is charged through the small thermal resistance of the mirror legs. Additionally, the bigger size of the microcantilever capacitor reduce the electrical resistance of the loop and therefore decrease heat dissipating in the structure. In this way, in comparison to our previous design, the mentioned above measures reduce the influence of the thermal phenomena on the microcantilever parasitic deflection.

The parameters of the microcantilever were determined based on thermal noise analysis according to the equipartition of energy theorem^26^. Thermal noise was recorded by a SIOS Nano Vibration Analyzer and analyzed with in-house-written software for spectral analysis. The microcantilever’s resonant frequency was measured to be equal to ${f}_{r}=6.254 \, \text{kHz}$, and the quality factor $Q=26$. The stiffness was ${\text{k}} = 0.15\;\frac{{\text{N}}}{{\text{m}}}$. The resistance of the microcantilever was also measured: $R=4.2 \, {\text{k}}\Omega$.

The product of the magnetic field and length of the path on the microcantilever was determined in the same setup. The microcantilever was actuated with a defined current; vibrations of the microcantilever were registered with SIOS NanoVibration Analyzer.

To verify the theoretical description of the microcantilever’s parameters, quasi-static actuation was performed. The microcantilever was with frequency much smaller than the resonant frequency. In this case the cantilever deflection was induced only by electromagnetic force. This result was compared with the theoretical description of force. The measured deflection amplitude was $y=90\, \text{nm}$. This makes the measured electromagnetic force F_E-m_ equal to:

8 $$F_{{E{\text{ - }}m}} = y \times k = 90\;{\text{nm}} \times 0.15\;\frac{{\text{N}}}{{\text{m}}} = 13.5\;{\text{nN}}.$$

On the other hand, the electromagnetic force can be calculated basing on the cantilever geometry, current and magnetic induction. The length of the microcantilever active path (Fig. 6b) was *l* = 100 μm, the current was set to be $I=1 \, \text{mA}$, and the magnetic induction measured with a Gaussmeter was $B=135 \, \text{mT}$. The magnetic field and current direction are assumed to be perpendicular. Thus, the calculated force F_E-c_ is:

9 $${F}_{E-c}=I\times B\times l=100 \, \upmu\text{m}\times 135 \, {\text{mT}}\times 1 \, {\text{mA}}=13.5 \, \text{nN}.$$

During electromagnetic actuation, Joule heating in the body of the microcantilever is present. The deflection of the bilayer is proportional to the power of the electric current. Current of 1 mA dissipates in the pfMEMS power of 4.2 mW. The power amplitude of electrothermal actuation was 4 orders of magnitude lower than that of electromagnetic actuation and equal to the effect of a force of 116.4 pN. Therefore, the influence of the electrothermal actuation can be neglected.

Thermomechanical actuation is also induced by an absorbed laser beam. The microcantilever presented in this article was equipped with a gold mirror. According to Ref.^23^, for a wavelength of 1310 nm, gold has a reflectance of *R* = 97.46%.

Thermally induced deflection is not sensitive to the direction of the active force. However, optomechanical actuation would cause deflection according to the direction of the photon force. This means that static deflection should be side-sensitive. The given data are sufficient to determine the expected values of thermal and optomechanical deflection. From 4.2 mW of radiation, approximately 97.5% is reflected, which actuates a mirror-like surface with the following force:

10 $${F}_{p}=\frac{97.5\% \times 2\times 0.0042}{c}=27.3\, \text{pN}.$$

The thermal effect is proportional to 2.5% of the optical power and can be estimated experimentally for 1.5 mW actuation:

11 $${F}_{th}=2.5\%\times 116.4\, \text{pN}=2.9 \,\text{pN}.$$

It should be noted that as both thermal and optical deflections are proportional to the optical power, the relation of forces should remain constant regardless of the optical power. The ratio of the electrothermally induced force and therefore excited optomechanically is 10%.

## Data Availability

The datasets generated and/or analysed during the current study are available in the Google Drive repository, https://drive.google.com/drive/folders/1L11GQo9lIl6J_lHXfBxXzshh45LjhPVq?usp=sharing.
